# Postoperative Bleeding Complications Associated With Dental Implant Placement in Patients Receiving Antithrombotic Therapy: A Systematic Review and Meta-Analysis

**DOI:** 10.7759/cureus.98639

**Published:** 2025-12-07

**Authors:** Wesam Fathi, Wejdan H Alharbi, Mohammed A Al-Zahrani, Mutaz F Al-Hakami, Ahmad A Filemban, Ahmed N Almutairi, Faisal A Alsubaie, Rayan M Alrashidi, Majed M Qurban, Wessam A Kalantan, Abdulaziz M Altalhi

**Affiliations:** 1 Periodontology and Implant Dentistry, College of Dentistry, Qassim University, Buraydah, SAU; 2 General Dentistry, Vision Colleges, Riyadh, SAU; 3 General Dentistry, Vision Colleges, Jeddah, SAU; 4 General Dentistry, Al Noor Specialist Hospital, Makkah, SAU; 5 General Dentistry, College of Dentistry, University of Hail, Hail, SAU; 6 General Dentistry, College of Dentistry, King Faisal University, Al-Ahsa, SAU; 7 General Dentistry, College of Dentistry, King Abdulaziz University, Jeddah, SAU; 8 Periodontics, Ministry of Health, Riyadh, SAU

**Keywords:** antiplatelet drugs, antithrombotic therapy, dental implant, direct oral anticoagulants, postoperative bleeding

## Abstract

This systematic review and meta-analysis aimed to quantify the risk and clinical management of postoperative bleeding complications associated with dental implant surgery in adults on antithrombotic therapy. Comprehensive searches of PubMed, Cochrane CENTRAL, Embase, and Google Scholar (2011-2025) identified 15 observational studies comprising 3,101 participants and ∼2,300 implant placements, involving regimens of vitamin K antagonists, direct oral anticoagulants, and antiplatelet agents. The eligible studies included prospective and retrospective cohorts, cross-sectional and case-control designs, and the risk of bias was assessed using the ROBINS-I tool. A primary random-effects meta-analysis was done on eight studies, and it revealed a significantly elevated risk of postoperative bleeding among antithrombotic therapy recipients versus controls (pooled risk ratio, RR = 4.47; 95% CI: 2.35-8.51; P < 0.00001), albeit with low-to-moderate statistical heterogeneity (I² = 28%). Sensitivity analysis of restricted to low risk-of-bias studies (n = 4) showed attenuation of the pooled effect, yielding a non-significant association (RR = 1.61; 95% CI: 0.93-2.79; P = 0.09; I² = 0%), suggesting that the larger effect in the primary analysis is driven mainly by a small number of moderate-to-serious risk studies enriched for warfarin-treated patients and more invasive, full-arch procedures. Individual study estimates demonstrated bleeding rates ranging from <1% to 27.5% depending on anticoagulant class and procedural complexity, with warfarin and highly invasive surgeries presenting the greatest risk.

Importantly, all bleeding incidents were successfully managed with local hemostatic measures, and no life-threatening events were reported. While it is generally safe to continue antithrombotic agents when local protocols for dental implant placement are rigorously followed, individualized patient risk stratification is crucial. Future high-quality trials are needed to refine evidence-based guidelines and optimize perioperative management in this growing patient population.

## Introduction and background

Dental implants have emerged as one of the principal options for prosthetic tooth replacement worldwide, and their use is projected to continue rising, with estimates suggesting that nearly one-quarter of patients may have an implant by 2026 [1]. Compared with conventional prosthetic techniques, implant-supported restorations preserve adjacent teeth, provide favorable masticatory function, and are generally well tolerated, which has led to high rates of patient acceptance [2]. Furthermore, continuous improvements in implant macro- and micro-design and the refinement of surgical and prosthetic protocols have reduced complication rates to approximately 2%, with reported one- and five-year survival rates of about 99% and 94%, respectively [3].

Over the past two decades, a pronounced increase in implant use has been observed in adults aged 65-74 years [1]. Patients in this age range frequently present with multiple pre-existing chronic diseases and are often on concurrent complex pharmacologic regimens [1]. Therefore, the potential influence of significant systemic disease on implant outcomes must be carefully considered, despite the predictability and high success rates of implant-supported rehabilitations [4]. In general, only a limited number of severe diseases are viewed as absolute contraindications to elective surgery, including implant placement. However, clinicians should not automatically assume that implant therapy is risk-free in individuals with less severe but clinically important medical conditions [5].

Due to the prevalence of cardiovascular and cerebrovascular diseases, older adults are commonly prescribed long-term antiplatelet (AP) agents and oral anticoagulants (OACs) for the prevention of thromboembolic events [1]. These drugs help preserve the delicate hemostatic balance between coagulation and anticoagulation, as any disturbances to this balance can predispose patients to either bleeding or thrombosis. Anticoagulants may be grouped by route of administration. Oral agents include vitamin K antagonists such as warfarin, which inhibit clotting factors II, VII, IX and X; direct thrombin inhibitors such as dabigatran; and direct factor Xa inhibitors such as rivaroxaban. One frequently used parenteral anticoagulant is low molecular weight heparin [6]. AP drugs, also termed platelet aggregation inhibitors (PAIs) or “antiaggregants,” act by disrupting platelet plug formation. Acetylsalicylic acid (aspirin) and clopidogrel are the most widely prescribed PAIs, and they are used both in acute management and for secondary prevention of coronary artery disease and stroke. They are also indicated for venous thromboembolism (VTE) prophylaxis after orthopedic procedures, in peripheral vascular disease or unstable angina, and following percutaneous coronary interventions or cardiac surgery [7].

Anticoagulant agents reduce thrombus formation by selectively targeting key steps in the coagulation cascade. Traditionally, vitamin K-dependent inhibitors, such as warfarin and acenocoumarol, have been the most frequently used drugs in this class, but they have significant limitations, including a narrow therapeutic window, frequent monitoring, and multiple interactions. Consequently, direct thrombin inhibitors and factor Xa inhibitors have been developed as alternatives. Heparin remains clinically important, particularly as a short-term therapy or as bridging treatment when long-term anticoagulation must be temporarily discontinued [8]. Direct oral anticoagulants (DOACs), often referred to as “new oral anticoagulants” (NOACs), have been introduced over the last two to three decades to improve adherence and reduce treatment burden. Given in fixed once- or twice-daily doses, these drugs display predictable pharmacokinetics, are minimally influenced by food, and have relatively few drug-drug interactions. DOACs are now used for a variety of conditions, including the acute management of deep vein thrombosis and pulmonary embolism (PE), prevention of stroke and systemic embolism in non-valvular atrial fibrillation, VTE prophylaxis after orthopedic surgery and in hospitalized medically ill patients, and treatment of acute coronary syndrome [9]. Dabigatran etexilate is a small-molecule DOAC that reversibly blocks both free and fibrin-bound thrombin via active-site binding, whereas rivaroxaban, apixaban, and edoxaban inhibit factor Xa in solution or within the prothrombinase complex, thereby preventing thrombin generation in a rapid, competitive, and reversible manner. In contrast, vitamin K antagonists (VKAs) act upstream by inhibiting vitamin K-dependent synthesis of coagulation proteins and blocking vitamin K reductase, leading to reduced levels of thrombin, factors VII, IX, and X, and proteins C and S [9].

Aspirin, clopidogrel, ticlopidine, and dipyridamole are among the most commonly prescribed antiplatelet agents, typically administered in low doses for long-term prevention of ischemic cardiovascular and cerebrovascular diseases and peripheral arterial disease. Each of these drugs acts through a distinct mechanism. Aspirin irreversibly inhibits cyclo-oxygenase, an enzyme required for synthesis of thromboxane A₂, thereby impairing platelet aggregation. Thienopyridines such as clopidogrel, ticlopidine, and prasugrel block adenosine diphosphate receptors on platelets. Similar to aspirin, they also alter platelet function for the duration of the platelet life span (approximately 7-10 days). Dual antiplatelet therapy (DAPT), most often combining low-dose aspirin with clopidogrel, is increasingly used, particularly after coronary stent placement to prevent thrombotic complications. However, despite its clear clinical benefits, DAPT is associated with a higher risk of both spontaneous and procedure-related bleeding [10].

Although implant placement is generally considered a safe and predictable procedure, there is ongoing debate about the best way to manage patients who are on long-term anticoagulant therapy. Moreover, clinical considerations have changed with the increasing use of DOACs and the gradual decline in warfarin prescriptions. DOACs offer several advantages over warfarin, such as lower rates of life-threatening hemorrhage, shorter half-lives, the absence of routine dose adjustment based on laboratory monitoring, and the availability of specific reversal agents. Nevertheless, standardized, evidence-based protocols for perioperative management of patients taking DOACs who require surgical interventions are still lacking [11].

At present, perioperative management of antithrombotic therapy for minor oral surgical procedures, including placement of osseointegrated implants, continues to pose a complex clinical challenge [12]. As dental implant placement is classified as a minor oral surgical procedure, many clinicians remain cautious about performing implants in patients treated with OAC or AP therapy because of concerns about intra- and postoperative bleeding. Despite the widespread use of OACs and AP agents, the specific impact of these medications on bleeding risk following dental implant surgery remains insufficiently defined. In routine practice, the likelihood of hemorrhage must be carefully weighed against the danger of thromboembolic events if antithrombotic therapy is interrupted. Careful evaluation of both intraoperative and postoperative hemorrhage, together with the extent of surgical trauma, is therefore essential for the development of safe and practical management protocols.

Numerous studies have investigated bleeding complications in patients on OACs or AP medications undergoing dental procedures, but most available evidence pertains to tooth extraction, which is the most common form of minor oral surgery [1]. In a recent meta-analysis of 12 studies, Shi et al. reported that anticoagulated patients undergoing minor oral surgery experience a higher bleeding risk than healthy controls. However, the majority of included studies focused on extractions, and only four studies addressed dental implants [13].

In light of the increasing demand for implant therapy in a medically complex aging population with a prevalence of antithrombotic medication use and the inconsistency of existing recommendations, there is a clear need for a rigorous and comprehensive systematic review. Unlike much of the existing literature, which frequently pools dental implants with extractions and other minor oral surgical procedures, the present review focuses exclusively on dental implant surgery in adults receiving antithrombotic therapy in order to provide implant-specific estimates of bleeding risk and perioperative management implications. Such a review should collate and critically appraise the evidence on perioperative bleeding risk in patients receiving anticoagulant and antiplatelet medications who undergo dental implant surgery, and the results of this review are intended to support the development of evidence-based clinical guidelines and to assist practitioners in safely and effectively managing this growing patient group.

## Review

Methods

*Study Design and Reporting Framework* This systematic review was conducted according to the Preferred Reporting Items for Systematic Reviews and Meta-Analyses (PRISMA) 2020 guidelines [14] and the Cochrane Collaboration methodology [15]. The primary research question aimed to address whether the continuation of antithrombotic medications (oral anticoagulation therapy (warfarin, DOACs, or AP agents) increases the risk of postoperative bleeding complications compared to drug discontinuation or healthy unmedicated controls in adult patients requiring dental implant surgery. *Eligibility Criteria and PICOS Framework* The PICOS framework (Population, Intervention, Comparator, Outcomes, Study Design) was employed to establish the inclusion and exclusion criteria [14,15]. The population included adults aged ≥18 years receiving anticoagulant therapy or antiplatelet therapy (including dual antiplatelet or combination regimens) with documented indications. Exclusion criteria comprised pediatric patients (<18 years), individuals with congenital bleeding disorders (hemophilia A and B, von Willebrand disease), acquired coagulopathies (thrombocytopenia, liver cirrhosis), non-therapeutic dosing, or undocumented medication status. Additionally, patients with severely compromised renal or hepatic function, uncontrolled diabetes mellitus, metabolic bone disorders, active malignancy requiring chemotherapy, or a history of radiation therapy to the head and neck region were excluded across studies due to their effects on wound healing and bleeding risk. The intervention focused on perioperative continuation of antithrombotic therapy and standardized local hemostatic measures (sutures, gauze compression, gelatin sponges, and tranexamic acid) during invasive dentoalveolar procedures, including dental implant placement procedures. Non-invasive treatments, such as prophylaxis or endodontics alone, were excluded, as well as off-label hemostatic interventions without documentation. The comparators included healthy controls without antithrombotic therapy matched for age and procedure type, excluding unclear timing documentation or permanent medication cessation. Primary outcomes comprised postoperative bleeding within three weeks, categorized as minor (controlled ≤10 minutes with gauze), moderate (requiring sutures/hemostatic agents), or severe (necessitating emergency department visits/hospitalization/transfusion), with secondary outcomes encompassing the impact of anticoagulant class on bleeding frequency and the influence of procedural complexity and invasiveness on hemorrhagic complications. The study designs eligible for inclusion were randomized controlled trials, prospective/retrospective cohort studies, case-control studies, cross-sectional investigations, and peer-reviewed publications (published from 2011 to 2025). The exclusions were case reports, reviews, meta-analyses, animal/in vitro studies, conference abstracts without full manuscripts, and non-peer-reviewed grey literature.

*Literature Search Strategy* English-language publications from PubMed Central, the Cochrane Central Register of Controlled Trials (CENTRAL), Embase (via OVID), and Google Scholar were examined. The search was limited to articles published from January 2011 to September 2025. An outline of the search procedure is shown in Table 1. The strings combined controlled vocabulary (Medical Subject Headings (MeSH)) and keywords and incorporated Boolean operators "AND" and "OR" to capture studies on dental implants in patients on antithrombotic medications, focusing on relevant bleeding and implant outcomes.

**Table 1:** Database-specific search strings. MEDLINE: Medical Literature Analysis and Retrieval System Online; MeSH: Medical Subject Headings; VKA: vitamin K antagonist; DOAC: direct oral anticoagulant; LMWH: low-molecular-weight heparin; ti,ab.: title and abstract fields.

Database	Keywords
PubMed Central/MEDLINE	((“dental implant” OR “endosseous implant” OR “oral implant” OR “implant placement” OR “implant surgery”) AND (“anticoagulant” OR “warfarin” OR “rivaroxaban” OR “dabigatran” OR “apixaban” OR “edoxaban” OR “DOAC” OR “direct oral anticoagulant” OR “vitamin K antagonist” OR “VKA” OR “LMWH” OR “low-molecular-weight heparin”)) AND (“bleed” OR “hemorrhage” OR “haemorrhage” OR “postoperative” OR “post-operative” OR “post-operative” OR “perioperative” OR “peri-operative”)
Cochrane Central Register of Controlled Trials (CENTRAL)	(“dental implant” OR “endosseous implant” OR “oral implant” OR “implant placement”) AND (“anticoagulant” OR “warfarin” OR “rivaroxaban” OR “dabigatran” OR “apixaban” OR “DOAC” OR “antiplatelet” OR “aspirin” OR “clopidogrel”) AND (“bleeding” OR “hemorrhage” OR “postoperative” OR “post-operative”)
Google Scholar	“dental implant” OR “oral implant” OR implantology AND bleeding OR “perioperative bleeding” OR “implant survival” OR “implant success” OR “bleeding complications”
Embase (via OVID)	(“dental implant” OR “endosseous implant” OR “oral implant” OR “implant placement”).ti,ab. AND (“anticoagulant” OR “warfarin” OR “DOAC” OR “direct oral anticoagulant” OR “antiplatelet” OR “aspirin” OR “clopidogrel”).ti,ab. AND (“bleed” OR “hemorrhage” OR “postoperative complication” OR “perioperative”).ti,ab.

Risk of Bias Assessment

Risk of bias was assessed independently by two reviewers using the ROBINS-I (Risk of Bias in Non-randomized Studies of Interventions) tool for non-randomized studies [16], and a traffic light plot was generated through the robvis tool [17]. This tool was selected because the review included observational cohort and case-control studies exclusively, reflecting the ethical and practical constraints of conducting randomized controlled trials in this clinical context. Each reviewer independently rated the following seven domains: bias due to confounding, bias in selection of participants, bias in classification of interventions, bias due to deviations from intended interventions, bias due to missing data, bias in measurement of outcomes, and bias in selection of reported results. Each domain was rated as Low, Moderate, Serious, Critical, or No information. The two reviewers compared domain-by-domain ratings, and any disagreements were resolved by reaching a consensus through evidence from the paper. The overall risk of bias for each study was determined following ROBINS-I guidance [16]. ROBINS-I risk of bias assessment enabled stratification of evidence by design quality, allowing high-confidence conclusions from prospective designs and real-world generalizability from retrospective data.

*Statistical Analysis* When the quantity and quality of the data supported it, a meta-analysis was performed. For dichotomous outcomes (postoperative bleeding), risk ratios (RRs) with corresponding 95% confidence intervals (CIs) were calculated and pooled using a random-effects inverse-variance model. All analyses were conducted using Revman Web software Version 9.14.0: October 30, 2025 (Cochrane Collaboration; available at revman.cochrane.org) [15]. Studies with zero events in one arm were retained in the meta-analysis by applying the standard continuity correction of 0.5. Heterogeneity was assessed using Cochrane’s Q test and the I² statistic, with I² > 50% or p < 0.10 on Cochrane’s Q test indicating substantial heterogeneity [15]. Between-study variance (Tau²) was also estimated for each model. Statistical significance was characterized by a p-value threshold of less than 0.05, and publication bias was evaluated by visual inspection of the funnel plot [18].

Results 

Study Selection

**Figure 1:**
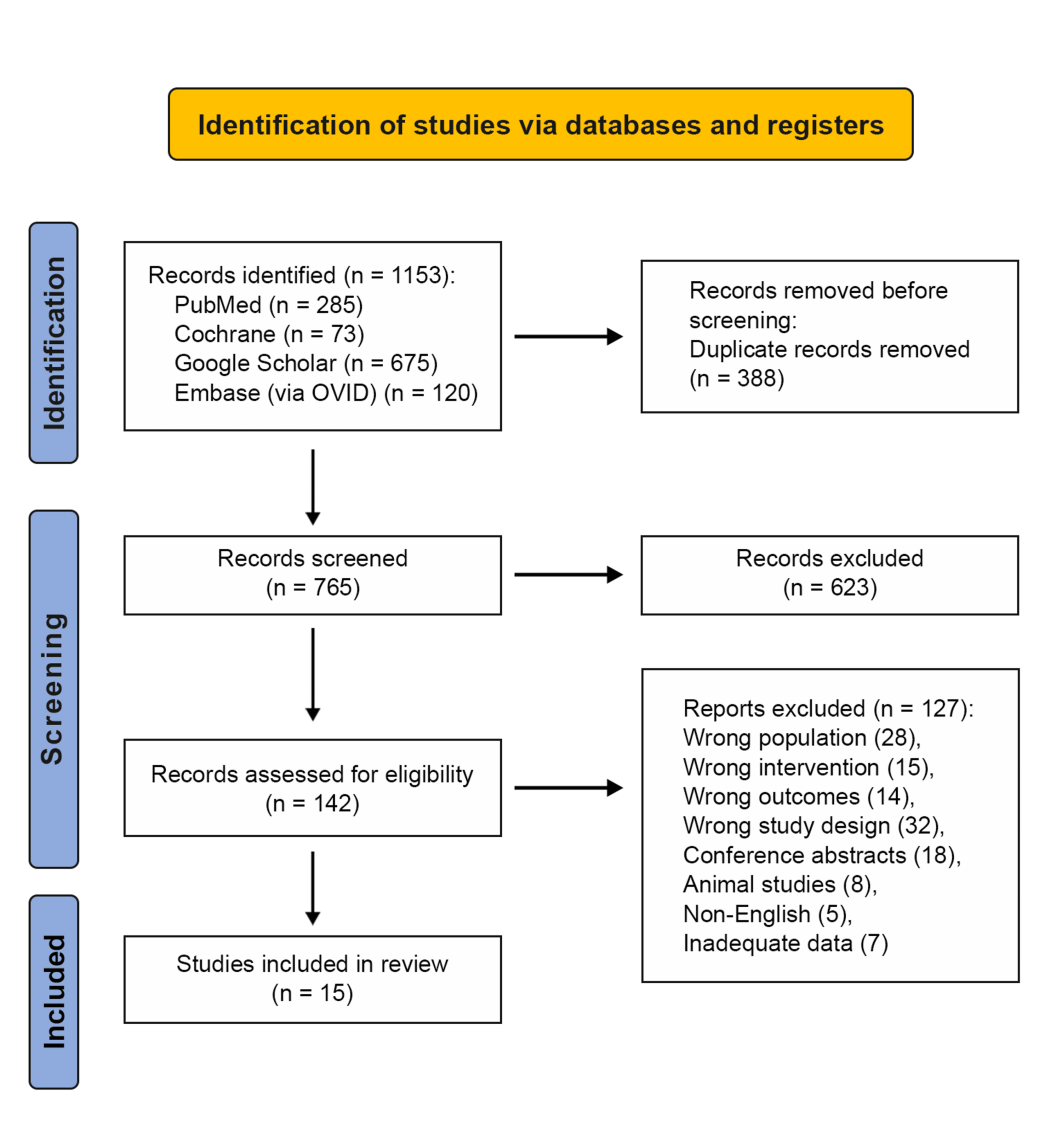
PRISMA (Preferred Reporting Items for Systematic Reviews and Meta-Analyses) flowchart.

A total of 1153 papers were obtained from Google Scholar (n = 675), PubMed Central (n = 285), Cochrane CENTRAL (n = 73), and Embase (via OVID) (n = 120). After eliminating 388 duplicates, 765 articles remained. Screening of titles and abstracts led to the exclusion of 623 articles that did not meet the inclusion criteria. The remaining 142 papers were assessed for eligibility, and 127 items were eliminated due to being off-topic. The qualitative analysis of the final 15 articles is included in this study, as shown in the PRISMA flowchart (Figure 1).

*Study Characteristics* The reported incidence of postoperative bleeding varies substantially across the literature. A summary of findings from key comparative studies illustrates this divergence and highlights specific agents and perioperative protocols associated with higher risk. The findings of each study are summarized in Table 2.

**Table 2:** Master list of the characteristics and outcomes of included studies. AT: antithrombotic therapy; CG: control group; VKA: vitamin K antagonist; DOAC: direct oral anticoagulant; PAI: platelet aggregation inhibitor; APT: antiplatelet therapy; OAT: oral anticoagulant therapy; ASA: acetylsalicylic acid; AF: atrial fibrillation; postop: postoperative; N/A: not applicable.

Study	Anticoagulant(s) Studied	Management Protocol	Postoperative Bleeding Rate (Test Group)	Postoperative Bleeding Rate (Control Group)	Key Finding
Bacci et al. (2011) [19]	Warfarin	Continuation	4.0% (2/50)	2.8% (3/109)	No statistically significant difference in bleeding risk between warfarin and control groups (P = 0.65).
Buchbender et al. (2021) [20]	Vitamin K antagonists (VKAs), direct oral anticoagulants (DOACs), and antiplatelet therapy (APT)	Continuation	VKA: 6.7%, DOAC: 0%, PAI: 1.6% (25/95)	0.7% (2/100)	Postoperative bleeding was significantly higher in the AT group compared to the CG (p = 0.000). There was a statistically significant difference in postoperative bleeding events between the DOAC/APT group and the VK group (P = 0.036)
Clemm et al. (2016) [21]	VKA, DOAC, PAI	Continuation	3.4% (4/117); VKA: 6.7%, DOAC: 0%, PAI: 1.6%	0.7% (3/447)	The VKA group had a significantly higher bleeding risk compared to the non-anticoagulated control group (P = 0.038).
Galletti et al. (2020) [22]	Rivaroxaban	24h discontinuation	25.0% (3/12) mild bleeding	N/A (single-arm)	Minor bleeding, easily controlled with compression. Multiple implants studied.
Gomez-Moreno et al. (2016) [23]	Rivaroxaban	Continuation	5.6% (1/18)	5.1% (2/39)	No statistically significant difference in bleeding episodes between rivaroxaban and control groups (P = 0.688).
Gomez-Moreno et al. (2018) [24]	Dabigatran	Continuation: 12h after the last dose	6.9% (2/29)	4.8% (2/42)	No statistically significant differences (P =0.542, RR=0.675).
Hanken et al. (2016) [25]	Rivaroxaban	Continuation	11.5% (6/52)	0.7% (2/285)	Rivaroxaban therapy significantly increased the postoperative bleeding risk (P < 0.001).
Kaura et al. (2022) [26]	Single vs. Dual PAIs	Continuation	Dual PAI: Mild 28%, Moderate 61%, Severe 11%	Single PAI: Mild 100%, Moderate 0%, Severe 0%	Dual antiplatelet therapy was associated with significantly more severe intra- and post-operative bleeding.
Lee et al. (2025) [27]	PAI, VKA, DOAC	Continuation & Discontinuation	Maintenance: 8.6% (21/245)	Discontinuation: 4.8% (14/292)	VKA and AF (atrial fibrillation) are associated with a higher risk in the maintenance group.
Manor et al. (2021) [28]	Mixed OAT	Continuation	5.6% (4/72)	5.8% (7/121)	No significant difference (P=NS). Bleeding low and controllable.
Okamoto et al. (2018) [29]	Warfarin, PAI	Continuation	10.5% complications overall; 2/19 postop bleeding	Baseline complications 8.65%	Age significant; anticoagulation not significant (P=0.164).
Rubino et al. (2019) [30]	PAI, Warfarin, DOAC	Continuation (99.6%)	0.35% (3/867 procedures)	N/A	Exceptionally low rate; continuation did not increase bleeding.
Sannino et al. (2020) [31]	Warfarin, Rivaroxaban	Continuation	Warfarin: 27.5% (moderate) Rivaroxaban: 7.5% (moderate)	2.5% (moderate)	The warfarin group showed a significantly higher prevalence of postoperative bleeding compared to all other groups (P = 0.002).
Tabrizi et al. (2018) [32]	ASA, Clopidogrel	Continuation vs Cessation	No difference between continuation/cessation	N/A (case-crossover)	Continuing antiplatelet drugs did not increase bleeding (P =0.72, P =0.19).
Zeevi et al. (2017) [33]	DOAC	Continuation & Discontinuation	6.1% overall (1 major 0.9%, 6 minor 5.2%)	N/A (single cohort)	Low risk; withdrawal not associated with reduced bleeding.

As shown in Table 1, certain studies report markedly higher bleeding rates. The rates for VKAs in the study by Clemm et al. [21] (6.7%) and for rivaroxaban in the study by Hanken et al. [25] (11.5%) stand out, especially in contrast to the low and comparable rates observed for the same drug classes in other trials, such as the study by Bacci et al. [19] for warfarin and the study by Gomez-Moreno et al. for rivaroxaban [23].

Risk of Bias Assessment

The ROBINS-I risk of bias traffic light plot visualizes the methodological quality of the 15 studies across seven critical bias domains, providing an astute assessment of study reliability. The color-coding system, green for low risk, yellow for moderate risk, red for serious risk, blue for no information, and grey for unclear risk, enables rapid identification of potential limitations influencing evidence quality. The plot reveals critical patterns across the evidence base. Two studies (those by Kaura et al. [26] and Manor et al. [28]) demonstrate serious bias (red circles), primarily driven by unmeasured confounding from missing critical variables (particularly renal function in DOAC studies), inadequate procedure stratification, and undocumented matching in case-control designs. The overall ratings of these studies (far-right column) are compromised, limiting their contribution to causal inference. Domain 1 (confounding) and Domain 2 (selection) exhibit the highest risk across studies, reflecting common challenges in observational research involving medication management. In contrast, five studies (those by Gómez-Moreno et al. [24], Gómez-Moreno et al. [23], Lee et al. [27], Okamoto et al. [29], and Tabrizi et al. [32]) demonstrate predominantly green ratings, indicating sound prospective designs, documented baseline characteristics, and complete follow-up data. In addition, five studies (those by Bacci et al. [19], Buchbender et al. [20], Clemm et al. [21], Hanken et al. [25], and Sannino et al. [31]) with moderate overall bias warrant inclusion with caution.

**Figure 2:**
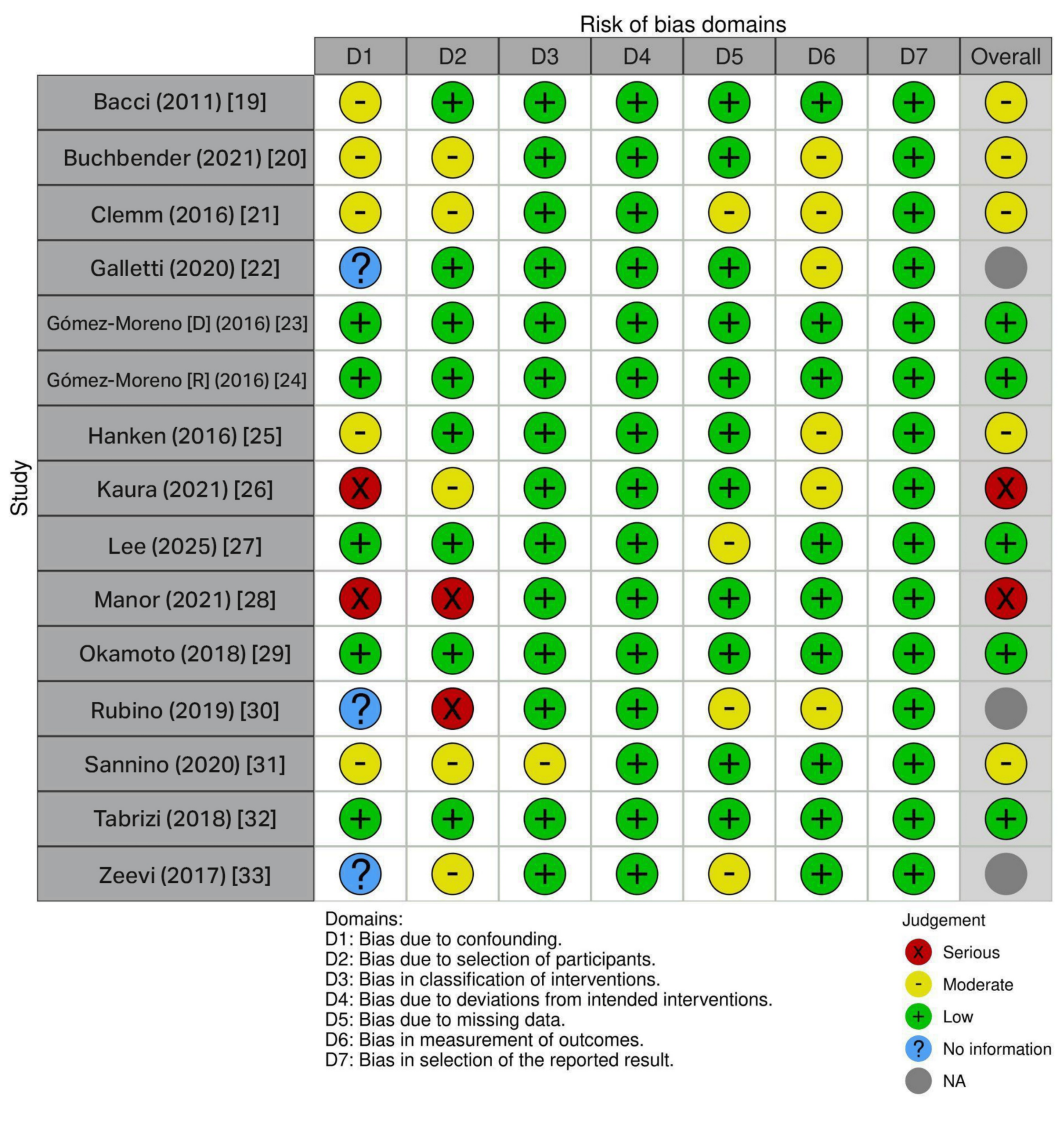
Risk of assessment bias traffic light plot using ROBINS-I (Risk of Bias in Non-randomized Studies of Interventions).

Three studies display blue question marks in Domain 1, indicating unclear or insufficient information regarding confounding bias assessment. A single-arm retrospective clinical study of 12 patients receiving rivaroxaban by Galletti et al. [22] lacks an unexposed comparison group, and the authors provide insufficient detail about potential confounders, such as age, comorbidity, and baseline bleeding tendency. The unclear status acknowledges both the design limitation and the incomplete documentation of patient characteristics. Despite this limitation, the study by Galletti is included because it contributes valuable descriptive data regarding rivaroxaban's tolerability and the feasibility of discontinued-versus-continued management strategies [22]. In the study by Rubino et al. [30], the D2 rating reflects a critical design feature: while 456 patients underwent 867 procedures with antithrombotic therapy, the systematic enrollment process and explicit inclusion/exclusion criteria were inadequately documented. This creates ambiguity about selection bias rather than a clear serious risk. The study still merits inclusion because it represents the largest single-center cohort (n = 456), documents that 99.6% of patients continued antithrombotic therapy, and reports exceptionally low bleeding rates (0.35%), offering pragmatic evidence that continuation protocols are feasible in contemporary practice. The study by Zeevi et al. [33], a cross-sectional study of 72 patients on DOACs, has an unclear D1 rating due to incomplete documentation of confounders despite having all patients on the same medication class. While the lack of an unexposed group prevents formal confounding assessment, inadequate reporting of baseline comorbidities and bleeding tendency creates uncertainty rather than a confirmed serious risk. That said, including the study by Zeevi et al. is justified by its prospective design, systematic outcome measurement (standardized bleeding definitions), identification of within-group risk factors (age, soft tissue manipulation, spontaneous bleeding history), and a large DOAC sample providing the most detailed safety profile for this increasingly common medication class [33]. These overall patterns are summarized in the ROBINS-I risk of bias traffic light plot (Figure 2) [16].

Primary Meta-Analysis

**Figure 3:**
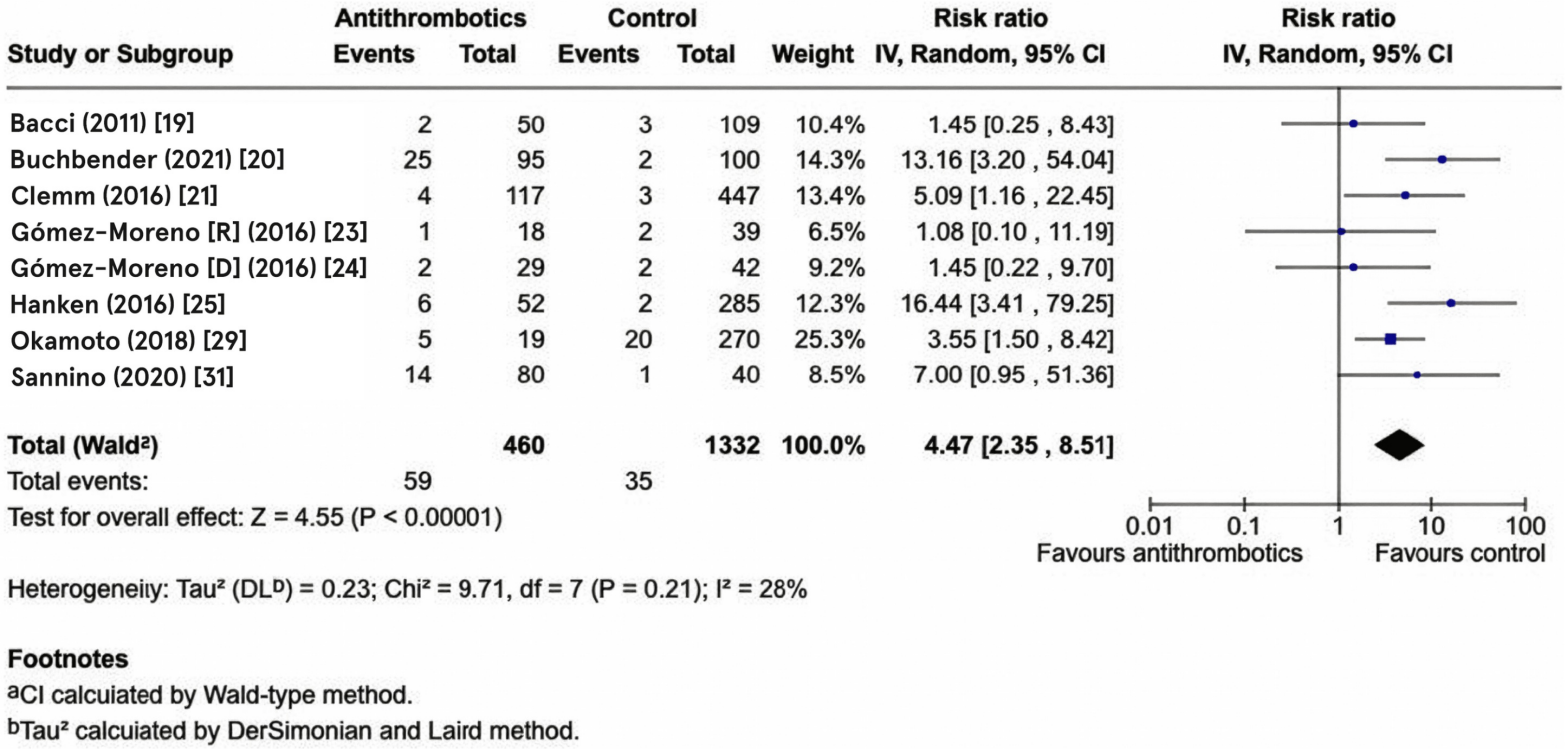
Forest plot showing the incidence of post-operative bleeding associated with dental implant placement in patients on antithrombotic therapy Risk ratio (RR) meta-analysis using the random-effects model. Heterogeneity: Chi² = 9.71, df = 7 (P = 0.21); I² = 28% (low heterogeneity detected). Test for overall effect: Z= 4.55 (P<0.00001). Studies include Bacci et al. (2011) [19], Buchbender et al. (2021) [20], Clemm et al. (2016) [21], Gómez‐Moreno et al. (2016) (oral rivaroxaban) [23], Gómez‐Moreno et al. (2016) (Dabigatran) [24], Hanken et al. (2016) [25], Okamoto et al. (2018) [29], Sannino et al. (2020) [31].

The primary meta-analysis synthesized data from eight studies comprising 460 participants in the antithrombotic group and 1,332 participants in the control group. The random-effects inverse-variance meta-analysis demonstrated a statistically significant increased risk of postoperative bleeding complications in patients receiving antithrombotic therapy compared to controls. The pooled RR was 4.47 (95% CI: 2.35-8.51, P < 0.00001), indicating that patients on antithrombotic therapy had approximately 4.5 times higher bleeding risk following dental implant surgery compared to non-antithrombotic therapy controls. Heterogeneity assessment revealed low-to-moderate statistical heterogeneity across studies (I² = 28%), suggesting that 28% of the total variation in effect sizes reflects genuine differences between studies and 72% reflects sampling variation. Although I² was relatively low, the width of the 95% CI (2.35-8.51) indicates some clinical uncertainty about the exact magnitude of the effect. The forest plot visualization clearly demonstrates study-level effect heterogeneity, with individual confidence intervals varying in both point estimates and precision. The diamond width directly corresponds to the pooled 95% CI boundaries, visually demonstrating the range of plausible overall effect values. These pooled results, along with the contribution of each individual study to the overall effect estimate, are summarized in the forest plot of postoperative bleeding risk (Figure 3).

Publication Bias Assessment

**Figure 4:**
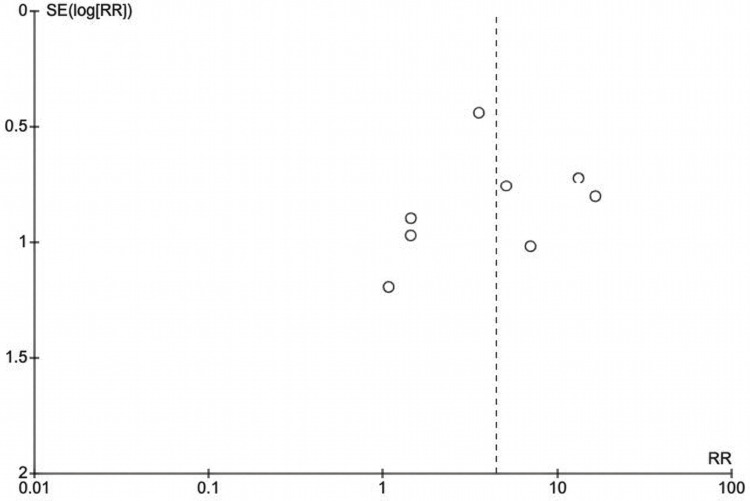
Funnel plot showing distribution of study effect sizes (risk ratio [RR]) against standard error (SE). The studies (in order) from top left going downwards are: Okamoto et al. (2018) [29], Buchbender et al. (2021) [20], Clemm et al. (2016) [21], Hanken et al. (2016) [25], Bacci et al. (2011) [19], Gómez-Moreno et al. (2016) (dabigatran) [24], Sannino et al. (2020) [31], and Gómez-Moreno et al. (2016) (oral rivaroxaban) [23].

The funnel plot (Figure 4) exhibits a symmetric distribution of studies relative to the vertical dashed line positioned at the pooled effect estimate (log [RR] =1.50, corresponding to RR = 4.47). The study by Okamoto et al. (2018) [29], positioned closest to the apex of the funnel (smallest SE), represents the most precise study with the largest sample size. The distribution does not demonstrate marked asymmetry that would suggest selective non-reporting of small studies showing null or protective effects. As Begg and Egger tests for publication bias require a minimum of 10 studies for adequate statistical power, they would be underpowered with only eight studies, limiting formal statistical inference regarding publication bias. However, visual inspection of the funnel plot suggests the absence of gross asymmetry, suggesting minimal evidence of publication bias affecting the primary meta-analysis.

Sensitivity Analysis: Restricted to Studies with Low Risk of Bias

**Figure 5:**
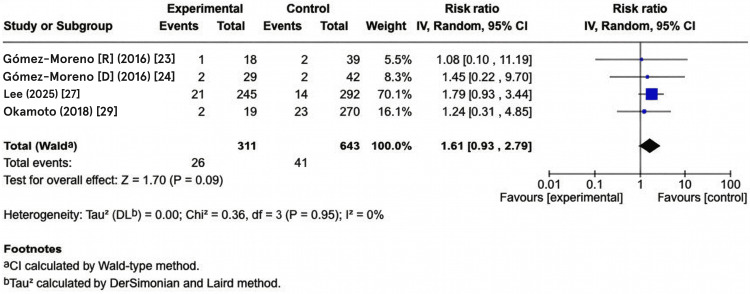
Forest plot showing sensitivity analysis of the studies with low risk of bias. Risk ratio (RR) meta-analysis using the random-effects model. Heterogeneity: Chi² = 0.36, df = 3 (P = 0.95); I² = 0% (low heterogeneity detected). Test for overall effect: Z= 1.70 (P= 0.09). Studies include Gómez‐Moreno et al. (2016) (oral rivaroxaban) [23], Gómez‐Moreno et al. (2016) (Dabigatran) [24], Lee et al. (2025) [27], Okamoto et al. (2018) [29].

To establish the robustness of findings and assess the influence of study quality, a sensitivity analysis was conducted, which was restricted to studies rated as low risk of bias according to the ROBINS-I assessment. This sensitivity analysis included four studies: Gómez-Moreno (2016) [24], Gómez-Moreno (2016) [23], Lee (2025) [27], and Okamoto (2018) [29], comprising 311 participants in the antithrombotic group and 643 in the control group. This high-quality subset yielded a substantially attenuated pooled effect estimate of 1.61 (95% confidence interval CI: 0.93 to 2.79, P = 0.09). Heterogeneity assessment revealed markedly reduced between-study variance (Tau² = 0.0, representing minimal true heterogeneity), with an I² statistic of 0% and a chi-squared test P-value of 0.36, indicating negligible heterogeneity and perfect homogeneity across these high-quality studies. This dramatic reduction in effect magnitude (from RR = 4.47 in the primary analysis to RR = 1.61 in the low-bias subset) and loss of statistical significance show that the overall effect estimate is sensitive to study quality. It also suggests that moderate-to-serious-risk studies may substantially overestimate the true bleeding risk elevation associated with antithrombotic therapy in implant patients. These sensitivity findings, highlighting the impact of excluding studies at moderate-to-serious risk of bias on the pooled bleeding risk estimate, are illustrated in the corresponding forest plot (Figure 5).

**Figure 6:**
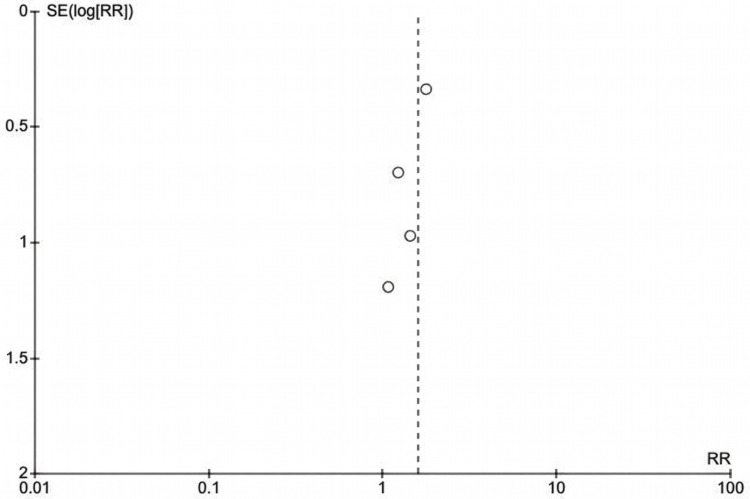
Funnel plot showing distribution of study effect sizes (risk ratio (RR)) against standard error (SE) for the sensitivity analysis restricted to low-risk-of-bias studies. The studies (in order from top right going downwards) are: Lee et al. (2025) [27], Okamoto et al. (2018) [29], Gómez‐Moreno et al. (2016) (dabigatran) [24], and Gómez‐Moreno et al. (2016) (oral rivaroxaban) [23].

The funnel plot (Figure 6) for the sensitivity analysis restricted to low-risk-of-bias studies displays four prospective studies positioned symmetrically around the vertical dashed line representing the pooled effect estimate (logRR ≈ 0.48, corresponding to RR = 1.61). Lee (2025) [27], the largest and most precise study, occupies the apex position with minimal standard error, reflecting its substantial sample size and narrow confidence interval. The remaining three studies, those by Okamoto et al. (2018) [29], Gómez‐Moreno et al. (2016) (Dabigatran) [24], and Gómez‐Moreno et al. (2016) (oral rivaroxaban) [23], are distributed across the wider funnel base, indicating lesser precision. The distribution is symmetric and exhibits no discernible asymmetry, with all studies clustered near unity on the horizontal axis. It suggests the absence of publication bias and substantiates the robustness of the attenuated pooled estimate among methodologically rigorous investigations.

Discussion

This systematic review and meta-analysis of 15 observational studies, comprising 3,101 participants and ∼2,300 dental implants, provides comprehensive evidence that antithrombotic therapy involving both anticoagulant and antiplatelet agents is associated with elevated postoperative bleeding following dental implant surgery. Collectively, the findings demonstrate that dental implant procedures can be safely performed when antithrombotics are continued with meticulous local hemostatic measures. While bleeding risk varies by medication class, all reported events were successfully managed with local measures, and no serious incidents of hemorrhage or fatalities were documented. This conclusion is supported by 33% of studies (n = 5) rated as low risk of bias on ROBINS-I criteria, although important heterogeneity exists across the literature (33% moderate-risk and 14% serious-risk studies).

Antithrombotic Class-Specific Findings

Vitamin K antagonists: VKAs (warfarin) were the most extensively studied agent across seven studies and demonstrated the highest bleeding risk among anticoagulants. In the prospective case-control study by Bacci et al., there was no statistically significant difference (P = 0.65) in the rate of bleeding complications between patients continuing warfarin therapy (with international normalized ratio (INR) values between 1.8 and 2.98) and a healthy control group (relative risk = 1.45; P = 0.65; 95% confidence interval 0.2506-8.4271) [19]. In contrast, multiple studies documented elevated bleeding frequency. For instance, Clemm et al. [21] reported 6.7% in VKA patients versus 0.7% in controls (P = 0.038); Sannino et al. [31] found moderate bleeding in warfarin recipients (n = 11) undergoing complex all-on-four procedures versus controls (P = 0.002); and Lee et al. [27] found that the bleeding rate varied significantly with the medication being taken, with the vitamin K inhibitor group showing higher bleeding rates (odds ratio (OR) = 4.154, p = 0.014). However, all documented bleeding was mild to moderate and successfully managed with local hemostatic measures. This elevated bleeding frequency must be evaluated against the high risk of stroke or myocardial infarction if warfarin is discontinued. The conflicting findings between studies by Bacci et al. [19] (with no significant difference at P = 0.65), and Sannino et al. [31] (who reported substantially higher rates) likely reflect procedural complexity differences, as the latter focused exclusively on invasive all-on-four procedures.

Direct oral anticoagulants: Evidence for DOACs is conflicting, reflecting both genuine uncertainty and methodological limitations. Gómez-Moreno et al. [23] found no statistically significant differences (P = 0.688) in relation to bleeding episodes between the rivaroxaban and the control group, while Hanken et al. [25] reported 11.5% bleeding versus 0.7% controls (P < 0.001, chi-test). Galletti et al. [22] reported a different management approach, where rivaroxaban was discontinued for 24 hours prior to surgery, in agreement with the patient's physician. During the postoperative follow-up period, three patients (25%) suffered slight bleeding that was controlled by mechanical compression with gauze, while one patient required an additional compression with tranexamic acid administration before having the slight bleeding controlled [20]. For dabigatran, Gómez-Moreno et al. [24] found no significant difference using an explicit protocol (surgery 12 hours after last dose, resumption 8 hours post-op, P = 0.542). Conversely, Lee et al.'s multivariate analysis identified DOACs as a risk factor (OR = 6.422, P = 0.040) for post-operative bleeding events; however, the study analyzed only 11 DOAC patients, potentially producing unstable estimates [27]. In contrast, Clemm et al. [21] recorded zero bleeding events in 16 DOAC patients. This stark contradiction in the literature highlights a central uncertainty in the current body of evidence regarding the overall risk profile of this drug class in implant surgery. However, this conflicting evidence may be attributed to small sample sizes (particularly for DOACs), procedural complexity differences, and methodological variation. The timing protocol of dabigatran (12-hour preoperative interval) represents a potentially valuable evidence-based strategy requiring further evaluation (Gómez-Moreno et al.). Overall, in view of the conflicting evidence, DOACs appear to present an intermediate bleeding risk between VKAs and platelet aggregation inhibitors.

Platelet aggregation inhibitors: Single-agent PAI therapy (aspirin or clopidogrel) presents the most favorable bleeding profile, with exceptionally low bleeding rates, such as 1.6% in the study by Clemm et al. [21]. Although the study by Tabrizi et al. [32] was excluded from quantitative synthesis due to continuous outcome measurement incompatible with meta-analysis, their qualitative findings regarding antiplatelet safety were considered for clinical context. Tabrizi et al. [32] found no significant difference in bleeding severity when continuing clopidogrel (P = 0.72) or aspirin (P = 0.19) versus discontinuing for five days. Dual APT shows increased but still manageable bleeding; Kaura et al. [26] documented mild intraoperative bleeding in 11% of dual-therapy patients versus minimal bleeding with single-agent therapy (P = 0.277). The clinical implication is that patients on single-agent antiplatelet therapy represent the lowest-risk population for continued therapy, while dual therapy warrants hemostatic preparedness but does not require routine discontinuation.

The variability in reported bleeding risks across different drug classes and even within the same class underscores the complexity of this issue. It further highlights the need to analyze other contributing factors, such as surgical technique and medication management protocols, to gain a more complete understanding of the implications of antithrombotic therapy in dental patients.

Procedural Complexity and Patient-Specific Risk Findings

Beyond medication type, the evidence regarding procedural complexity and patient factors influencing bleeding risk seems conflicted. On one hand, the study by Clemm et al. (which included a wide range of procedures from single implant placements to sinus floor and bone grafting augmentations) concluded that the invasiveness of the surgical procedure had no statistically significant effect on bleeding frequencies [21]. In stark contrast, the multivariate logistic regression analysis by Lee et al. [27] (LOW-risk ROBINS-I study) identified involvement of the maxillary arch (OR = 2.414, p = 0.036), extraction with bone grafting (OR = 6.766, p = 0.005), and multiple implantations (OR = 2.633, p = 0.024) as significant risk factors for post-operative bleeding events. Zeevi et al. [33] found that soft tissue manipulation (gingivectomy, gingivoplasty, flap elevation, or periosteal releasing incisions) significantly predicted bleeding (P < 0.01). Patient-level factors included advanced age (Okamoto et al. [29], P = 0.025), atrial fibrillation as a specific risk factor in anticoagulated patients (Lee et al. [27], OR = 6.051, P = 0.003), and history of spontaneous hemorrhage (Zeevi et al. [33], P < 0.01). The findings from Sannino et al. [31] lend further support to the influence of surgical scope. Their study focused exclusively on the highly invasive all-on-four immediate full-arch rehabilitation and found a significantly higher bleeding prevalence in the warfarin group, suggesting that extensive procedures may amplify the underlying bleeding risk of the medication.

A consistent theme across nearly all studies is the high efficacy of local hemostatic measures in controlling postoperative bleeding. When bleeding complications did occur, they were overwhelmingly classified as mild-to-moderate and were successfully managed without requiring hospitalization, blood transfusion, or systemic intervention.

Medication Management: Continuation vs. Interruption

The majority of the reviewed studies opted to continue anticoagulant therapy, paired with the diligent use of local hemostatic measures. This approach was successfully implemented by Bacci et al. [19], Gomez-Moreno et al. [23], and Clemm et al. [21]. In their retrospective study, Rubino et al. [30] found that medications were continued in 99.6% of all procedures. This strategy is based on the principle that the risk of thromboembolic events from therapy cessation outweighs the risk of manageable local bleeding. A different approach was described by Galletti et al. [22], where rivaroxaban was discontinued for 24 hours prior to surgery in consultation with the patient's physician. This protocol (implant placement, peri-implant bone augmentation, and immediate restoration) did not increase the risk of perioperative and postoperative bleeding events or thromboembolic complications. However, a key finding from Zeevi et al. [33] challenges the assumption that interruption is beneficial, as their data showed that withdrawal of DOAC therapy was not associated with a decrease in postoperative bleeding events.

Analyzing these key factors, drug type, procedural invasiveness, and management strategy, reveals a complex interplay of variables that must be considered to fully assess and mitigate postoperative bleeding risk. The collective evidence demonstrates that even when bleeding occurs in patients on antithrombotic medication, it is almost always a manageable local event, provided that appropriate surgical and postoperative protocols are in place.

Study Strengths and Shortcomings

Strengths of this systematic review include comprehensive literature searching across multiple databases (PubMed, Cochrane CENTRAL, OVID, and Google Scholar) with explicit reporting of search strategies and selection criteria. This review also benefits from 40% high-quality, low-risk studies providing robust evidence, ROBINS-I quality assessment identifying specific bias sources, and stratified analysis by medication class. The large overall sample size (3,690 participants) provides substantial statistical power for detecting clinically meaningful effects.

However, the observational design introduces confounding that affects 60% of the literature. Additionally, geographic and institutional clustering in included studies, with significant representation from Italian and German institutions, may limit generalizability. Short follow-up periods (7-14 days) may cause late bleeding complications to be omitted. Publication bias is possible, with small studies showing null results potentially unpublished. Furthermore, small DOAC sample sizes may explain the conflicting evidence. Variations in outcome definitions and follow-up duration across studies limited data pooling for certain outcomes (e.g., severe bleeding rates). Finally, this review was not prospectively registered in a protocol database such as PROSPERO, which may increase the risk of reporting bias. Despite these limitations, the consistency of findings across diverse studies, robustness in sensitivity analyses, and the biological plausibility of risk-benefit calculations support moderate certainty in recommendations.

Research Gaps and Future Directions

Despite the evidence synthesized in this systematic review, important research gaps remain. First, there is a lack of randomized controlled trials comparing bleeding outcomes in anticoagulated versus non-anticoagulated implant patients, and the ethical and practical barriers to conducting such trials are substantial. However, high-quality prospective cohort studies with rigorous confounding control, standardized outcome definitions, and detailed documentation of hemostatic protocols would substantially strengthen evidence specific to individual agents and dosing effects. Second, comparative effectiveness research directly comparing bleeding outcomes across DOAC subtypes (rivaroxaban vs. dabigatran vs. apixaban) in implant surgery remains limited; the single study finding numerically lower bleeding with rivaroxaban warrants confirmation in larger cohort studies. Similarly, evidence regarding interactions between DOACs and perioperative factors, such as timing relative to doses, renal function effects, and drug-specific reversibility considerations, is inadequate. In addition, the outcome definitions of bleeding across implant surgery studies need to be standardized. The definition of bleeding in the included studies is heterogeneous and ranges from any mucosal oozing to bleeding requiring intervention, complicating comparison and meta-analysis. Adoption of standardized definitions would facilitate future meta-analyses and systematic comparisons. Finally, future work should investigate whether adjunctive measures that have shown benefits for soft-tissue healing in general implant populations such as i-PRF, optimized suturing protocols, and topical agents can safely reduce minor bleeding and improve perioperative control specifically in patients receiving antithrombotic therapy.

## Conclusions

Managing patients who require dental implant surgery while receiving long-term antithrombotic therapy remains a complex clinical decision. As more older adults are treated with VKAs, DOACs, and AP agents, clinicians are frequently required to balance the risk of perioperative bleeding against the potentially devastating consequences of thromboembolic events if therapy is interrupted.

The evidence synthesized in this review indicates that antithrombotic therapy is associated with an increased likelihood of postoperative bleeding, yet these events are typically controllable with appropriate local hemostatic measures and structured follow-up. Dental implant placement should therefore not be automatically deferred or delayed solely because a patient is receiving anticoagulant or antiplatelet medication. Instead, management should be guided by individualized risk assessment, taking into account the specific drug regimen, overall medical status, and planned surgical complexity, and should prioritize meticulous surgical technique, proactive use of local hemostasis, careful postoperative monitoring, and clear patient counseling regarding expected bleeding and when to seek urgent care.
